# Fluorescence Correlation Spectroscopy Measurements of the Membrane Protein TetA in *Escherichia coli* Suggest Rapid Diffusion at Short Length Scales

**DOI:** 10.1371/journal.pone.0048600

**Published:** 2012-10-31

**Authors:** David Chow, Lin Guo, Feng Gai, Mark Goulian

**Affiliations:** 1 Department of Physics and Astronomy, University of Pennsylvania, Philadelphia, Pennsylvania, United States of America; 2 Department of Chemistry, University of Pennsylvania, Philadelphia, Pennsylvania, United States of America; 3 Department of Biology, University of Pennsylvania, Philadelphia, Pennsylvania, United States of America; Centre National de la Recherche Scientifique, Aix-Marseille Université, France

## Abstract

Structural inhomogeneities in biomembranes can lead to complex diffusive behavior of membrane proteins that depend on the length or time scales that are probed. This effect is well studied in eukaryotic cells, but has been explored only recently in bacteria. Here we used fluorescence recovery after photobleaching (FRAP) and fluorescence correlation spectroscopy (FCS) to study diffusion of the membrane protein TetA-YFP in *E. coli*. We find that the diffusion constant determined from FRAP is comparable to other reports of inner membrane protein diffusion constants in *E. coli*. However, FCS, which probes diffusion on shorter length scales, gives a value that is almost two orders of magnitude higher and is comparable to lipid diffusion constants. These results suggest there is a population of TetA-YFP molecules in the membrane that move rapidly over short length scales (∼ 400 nm) but move significantly more slowly over the longer length scales probed by FRAP.

## Introduction

Because biological membranes are two-dimensional fluids, their lipid and protein constituents diffuse within the plane of the membrane. When the fluid mosaic model of the cell membrane was first proposed, it was recognized that this lateral diffusion was likely to be critical for the modulation of membrane shape and the redistribution of proteins and lipids [1]. However, while early models of the membrane often envisioned proteins freely and homogeneously diffusing at all length scales, this has long been known not to be the case in eukaryotic cells [2]–[4]. Many membrane proteins exhibit complex diffusive behavior, reflecting structures in biological membranes. Cholesterol-enriched lipid microdomains or “rafts” can include or exclude proteins to varying degrees [5], [6], and scaffolding proteins can promote the clustering of membrane proteins [7]. Moreover, cytoskeletal structures interacting with membranes can compartmentalize phosopholipids, causing the lipids to hop intermittently from one domain to another, and resulting in measurements of diffusion constants that differ by a factor of ten, depending on the length scale probed [8].

In bacteria, the organization and mobility of membrane constituents are less well characterized. Measurements of diffusion in bacterial membranes have been limited at least in part due to the small size of most bacteria (∼ 1–2 µm), which can make measurements technically challenging [9], [10]. Diffusion constants in bacterial membranes have generally been determined from fluorescence recovery after photobleaching (FRAP) and single particle tracking experiments (see [2], [9] for recent reviews) and fall in the range 0.1–0.01 μm^2^/s for the cytoplasmic membrane [11]–[13] and the outer membrane (for Gram-negative bacteria) [2], [14]–[17]. Notably, these values are significantly slower than typical lipid diffusion constants [18]. Several studies have resolved mixed populations of fast and slow diffusing species or found evidence of confined diffusion, which suggest structural order in bacterial membranes that may depend on length scale and location in the cell [2], [11], [14]–[17], [19].

In this study, we measured the diffusion constant of the inner membrane protein TetA-YFP in live *E. coli* cells using two techniques, fluorescence recovery after photobleaching (FRAP) and fluorescence correlation spectroscopy (FCS), which probe diffusion on short and long length scales, respectively. While the FRAP value obtained here is comparable to diffusion constants of other membrane proteins in *E. coli*, the FCS measurement gives a diffusion constant that is higher by almost two orders of magnitude, and is comparable to lipid diffusion constants. This suggests that a population of TetA-YFP molecules have very high mobility at short length scales but are constrained to slower diffusion on longer length scales. We also observe similar behavior for a second membrane protein, Tar, suggesting the results may reflect an architecture in or adjacent to the inner membrane that hinders diffusion.

## Results and Discussion

**Figure 1:**
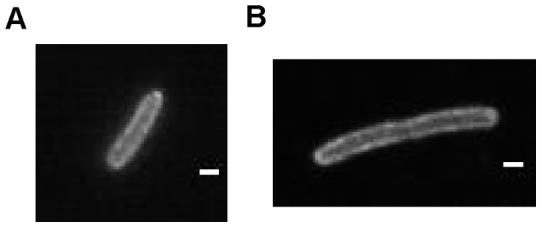
TetA-YFP fluorescence. Fluorescence images of cells expressing TetA-YFP. (A) A cell growing in minimal glucose medium. (B) A cell grown with cephalexin, causing filamentation, in order to perform FRAP measurements (see Methods). The scale bars indicate 1 µm.

TetA is an antiporter consisting of twelve transmembrane domains that pumps tetracycline out of the cell (across the *E. coli* inner membrane) in exchange for protons. To study the diffusion of TetA, we constructed a translational fusion of the gene for yellow fluorescent protein, *yfp*, to the 3′ end of *tetA* integrated at the phage lambda attachment site in the *E. coli* chromosome. The strain also contains a chromosomal copy of *tet* repressor, *tetR*, which prevents *tetA* transcription in the absence of tetracycline. When *tetA-yfp* transcription is induced, YFP fluorescence is localized to the edges of the cell, consistent with inner membrane localization for TetA-YFP (Figure 1A). The TetA-YFP fusion confers tetracycline resistance, indicating that it is functional as a tetracycline efflux pump.

To study the diffusion of TetA-YFP, we first turned to fluorescence correlation spectroscopy (FCS) [20]–[22]. FCS measures temporal correlations in fluorescence intensity fluctuations caused by one or more fluorescent molecules diffusing in and out of an illuminated excitation volume (∼400 nm across in our experiments). These correlations are quantified by the autocorrelation function *G*(τ):
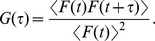



In the above expression, 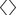 denotes a time average and *F*(*t*) is the fluorescence intensity at time *t*. *G*(τ) may be written explicitly in terms of the timescales of the dynamic processes causing the fluorescence fluctuations, such as diffusion, binding reactions, triplet state blinking and others (see Methods and [20]–[27]).

**Figure 2:**
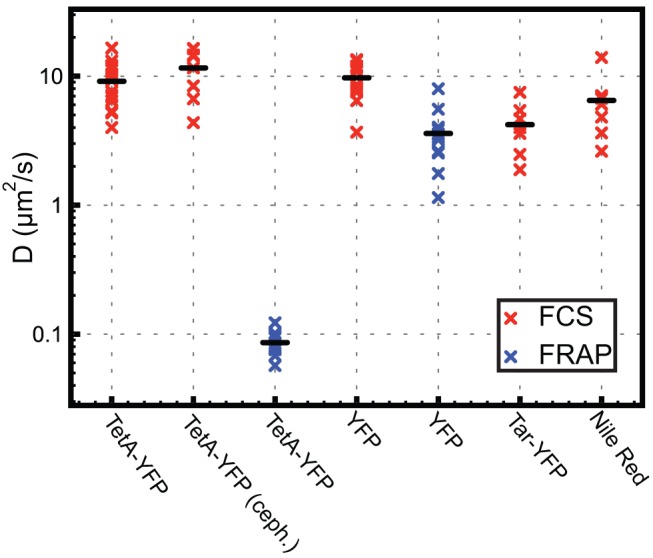
Diffusion constants. Diffusion constants measured with FCS (red symbols) and FRAP (blue symbols), from left to right: TetA-YFP measured by FCS in *E. coli* strain DGC103 (15 measurements, 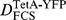 = 9.1±3.4 µm^2^/s; all values presented are mean ± SD); TetA-YFP measured by FCS in cephalexin-treated DGC103 (8 measurements, 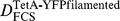 = 11.6±4.3 µm^2^/s); TetA-YFP measured by FRAP in DGC103 (12 measurements, 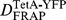 = 0.086±0.017 µm^2^/s); cytoplasmic YFP measured by FCS in *E. coli* strain DGC111 (13 measurements, 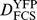 = 9.7±2.8 µm^2^/s); cytoplasmic YFP measured by FRAP in *E. coli* strain EPB255/pMG32 (12 measurements, 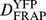 = 3.6±1.7 µm^2^/s); Tar-YFP measured by FCS in *E. coli* strain MC4100/pVS263 (7 measurements, 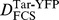 = 4.2±1.7 µm^2^/s); Nile Red measured by FCS in *E. coli* strain DGC102 (8 measurements, 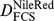 = 6.5±3.2 µm^2^/s).

Using FCS, we measured the diffusion constant of TetA-YFP in DGC103 and found 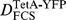 = 9.1±3.4 µm^2^/s (Figure 2). This value is surprisingly large, as it is comparable to lipid diffusion constants [18], [28]–[31] and roughly two orders of magnitude higher than reported diffusion constants for other membrane proteins in *E. coli* [9], [12], [13]. These previously reported diffusion constants were determined from fluorescence recovery after photobleaching (FRAP) experiments, however, and we are unaware of other FCS measurements of membrane protein diffusion in live *E. coli*. We also note that diffusion constants of membrane proteins and lipid probes in large unilamellar vesicles measured by FCS are comparable to our FCS values for the TetA-YFP diffusion constant in *E. coli* [23], [32].

For FRAP experiments, a region of interest within the cell is bleached. The subsequent diffusion of fluorescent molecules into the bleached region is then followed by measuring the spatial distribution of fluorescence as a function of time by fluorescence microscopy. This fluorescence recovery data is then fit to the expected solution of the diffusion equation. To compare with previous measurements of membrane protein diffusion in *E. coli*, which were made with FRAP, we also used this technique to measure the diffusion of TetA-YFP.

As in previous FRAP studies [12], [13], cells were made to form filaments by treating with cephalexin, an antibiotic that blocks septation (Figure 1B). We measured the diffusion constant of TetA-YFP in the strain DGC103 and found 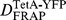 = 0.086±0.017 µm^2^/s (Figure 2), with mobile fraction *K* = 1.04±0.09. This value is similar to diffusion constants measured for other membrane proteins in *E. coli* [9], [12], [13], and markedly different from the value we obtained by FCS. The difference between the FRAP and FCS results is not due to cell filamentation since the diffusion constants from FCS were the same for cephalexin-treated and untreated cells (Figure 2).

To test whether the high FCS diffusion constant was unique to TetA, we measured the diffusion constant of a second membrane protein, the *E. coli* chemoreceptor Tar, by FCS. Tar is an integral membrane protein with two transmembrane domains that forms a trimer of dimers in the membrane [13], [33], [34]. FRAP measurements of a Tar-YFP translational fusion gave a diffusion constant of 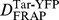 = 0.0171 µm^2^/s [13]. Using the same Tar-YFP fusion, expressed in a strain that does not produce other chemoreceptor proteins as in [13], we measured the diffusion constant by FCS to be 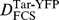 = 4.2±1.7 µm^2^/s (Figure 2). Thus the Tar diffusion constant determined by FCS is roughly two orders of magnitude higher than the diffusion constant determined by FRAP, which is similar to the results for TetA.

We also measured the diffusion constant of cytoplasmic YFP by both FCS and FRAP and found 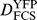 = 9.7±2.8 µm^2^/s and 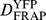 = 3.6±1.7 µm^2^/s, with K = 1.04±0.05 (Figure 2). Though not identical, both values are within the variation found for these methods in previous studies (e.g. [35], GFP-MinD and GFP; [36], CheY-GFP; [37], GFP; [12], GFP; [13], GFP; [38], GFP; [39], GFP). We also measured the diffusion constant of the fluorescent lipophilic dye Nile Red by FCS. For this measurement we used a strain (DGC102) that has a deletion in *acrB*, encoding a drug efflux pump that would otherwise pump Nile Red out of the membrane [40]. We found 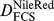 = 6.5±3.2 µm^2^/s, which is similar to FCS measurements of lipid probes in vesicles [23].

We note that FCS measurements on *E. coli* are subject to errors because their size, ∼1×2 µm, is near the size of the beam waist, ∼400 nm. At this scale, the membrane is not flat, but a surface which curves through the three dimensional space of the confocal detection volume. Applying a two-dimensional diffusion model as we have done (see methods) effectively projects some three dimensional motion into a plane, skewing the resulting measurements. Simulations suggest that the effect of this projection is an artificial confinement of the fluorophore, resulting in an underestimation of the true diffusion coefficient by 20–50% [9], [39]. While these corrections will affect the diffusion constants for TetA-YFP presented here, this source of error is unlikely to explain the two orders of magnitude that separate the diffusion constants determined by FCS and FRAP, and in fact would be expected to increase the difference between the two values. Other sources of error include photobleaching over the course of the experiment and deviations in the refractive index of the sample [9]. Based on the signal traces, however, photobleaching was minimal in our measurements. We also note that the agreement of the FCS measurements of cytoplasmic YFP diffusion with literature values and with our FRAP measurements suggests that these effects are unlikely to account for the large difference between the FCS and FRAP measurements of membrane protein diffusion.

A study of protein diffusion in mammalian cells also noted a difference between diffusion constants determined by FCS and FRAP for a membrane protein, the dopamine transporter, but not between those of a cytoplasmic protein, PICK1 [4]. In that case, the discrepancy was a factor of ten and it was suggested that the difference is due to the different length scales probed by the two techniques: the sampling radii of FRAP and FCS are different, and the techniques measure membrane proteins in very different environments. It was also demonstrated that disruption of lipid rafts significantly affects the diffusion constants.

In our experiments, and as has been noted in other studies [4], FCS and FRAP probe significantly different length scales. Our FCS observation volume had a diameter of ∼400 nm in the plane of focus whereas our FRAP experiments followed diffusion across the entire length of a filamented cell, ∼7 µm. The rapid diffusion that we observed from FCS experiments, in contrast with the slower diffusion that we and others have measured using FRAP [9], [12], [13], may therefore indicate an underlying structure in the membrane that affects protein mobility. This is further supported by single molecule tracking studies of the membrane protein TatA-YFP in *E. coli*, which provide evidence of at least two sub-populations with significantly different diffusion constants [9].

For eukaryotic cells, it has been suggested that domains or macromolecules within or adjacent to the membrane may form barriers to diffusion that are separated by distances greater than the short distance probed by FCS [41], [42]. Analogous structures in bacterial membranes may similarly restrict membrane protein mobility. Thus, membrane proteins may diffuse rapidly in over short length scales but, due to barriers or traps in the membrane, may exhibit a smaller effective diffusion constant over longer distances. Further work will be required to explore the architecture of the *E. coli* inner membrane to determine the precise mechanism that leads to variation in membrane protein mobility.

## Methods

The plasmids and strains used in this study are listed in Table 1.

**Table 1:** The plasmids and strains used in this study.

Strain	Relevant Genotype^a^	Induction/Treatment
DGC103	MG1655 *att_λ_*::[*tetR tetA*-*yfp cat*]	100 ng/mL atc (FRAP) or 100 ng/mL tc (FCS)
DGC111	MG1655 Δ*lacZ*::*yfp* FRT*-kan-*FRT	100 µM IPTG
EPB255	MG1655 Δ(*lacI lacZYA*)::FRT	none
DGC102	MG1655 Δ*acrB::*[FRT*-kan-*FRT]	2.6 nM Nile Red
MC4100^b^	*flhD*5301	none
**Plasmid**		
pMG32	pGFPmut3.1 Δ*gfp*::*yfp*	none
pVS263^c^	*tar*-*yfp*	none

a MG 1655 was from the *E. coli* Genetic Stock Center (Yale University, New Haven), CGSC# 7740.

b See [49], [50].

c See [13].

### Cell growth and preparation

Liquid cultures were grown at 37°C with aeration in minimal A medium [43] with 0.2% glucose and 0.1% Casamino acids. Minimal medium was chosen to minimize autofluorescence. Plasmids were maintained by growing with 50 µg/mL ampicillin. The *lac* promoter was induced using isopropyl-ß-D-thiogalactoside (IPTG) at 100 µM for single-copy YFP, and the *tet* promoter was induced using either tetracycline (tet) for FCS or anhydrotetracycline (atc) for FRAP, each at 100 ng/mL. Tar-YFP was expressed from the basal level of the uninduced Trc promoter. For FRAP measurements, and one of the FCS measurement sets on DGC103, cells were made to filament using 80 µg/mL cephalexin in the last 30 minutes of growth.

Agarose pads were made from 1% SeaKem LE Agarose (Cambrex, Rockland, ME) in minimal medium without a carbon source. For FRAP, 3 mL molten agarose was poured into 35 mm diameter coverglass-bottom petri dishes (Fluorodish, World Precision Instruments, Inc.). Cells were grown overnight to saturation, then diluted back 1∶500 into fresh medium with inducers if necessary (see above) and grown to mid exponential phase (optical density at 600 nm ≈0.2). 5 µL of this culture was placed under the agarose slab for imaging at 20°C. For FCS, cells were immobilized on an agarose pad placed between a microscope slide (Corning, Corning, NY) and number 1.5 cover glass (Corning, Corning, NY), essentially as described in [44]. Cells were grown as for FRAP, and measurements were made at 20°C.

### Strains and plasmids

The strain DGC103 was derived from AFS18 (A. Siryaporn and M. Goulian, unpublished), which contains the *tetR tetA* genes from Tn*10* integrated at the lambda attachment site in *E. coli* strain MG1655 [45]. DGC103 was constructed by lambda Red-mediated recombination using a DNA segment produced by PCR with the primers 5′-GCTCAGGGGAGTAAACAGGAGACAAGTGCTGGGCGTAAAGGAGAAGAACTTT-3′ and 5′-GGGCTGCAGGAATTCGATATCAAGCTTATCGTGTAGGCTGGAGCTGCTTC-3′ and the template pEB45, a derivative of pKD13 [46] in which *yfp* is upstream of and in the same direction as the kanamycin resistance gene. This DNA segment, which consists of *yfp* with flanking homology to the end of *tetA* and to the region downstream of *tetA* was recombined into AFS18 as described in [46] to create the translational fusion *tetA-yfp*. The construct was then moved into a clean MG1655 background by P1 transduction [43], and the *kan* resistance gene was removed via FLP recombinase as in [46]. DGC111 was constructed by using the upper primer 5′-TATGTTGTGTGGAATTGTGAGCGGATAACAATTTCACACAGGAAACAGCTATGCGTAAAGGAGAAGAACTTTTCACTGGA-3′ and lower primer 5′-CTCCAGGAGTCGTCGCCACCAATCCCCATATGGAAACCGTCGATATTCAGGTGTAGGCTGGAGCTGCTTCGAAGTTCCTA-3′ to create a product from pEB45 with flanking homology to *lacZ*, which was then recombined by the same method as above into MG1655, deleting *lacZ* and replacing it with *yfp kan*. This construct was moved into a clean MG1655 background by P1 transduction. EPB255 is MG1655 Δ(*lacI lacZYA*)::FRT. pMG32 is derived from pGFPmut3.1 (Clontech) with *yfp* in place of *gfpmut3.1* [47]. DGC102 was constructed by P1 transduction of Δ*acrB*::*kan* from the Keio collection knockout strain JW0451 [48] into MG1655. FCS measurements of Tar-YFP were made in the strain MC4100 [49], [50], which is *flhD* ^−^ and therefore does not express any chemotaxis proteins. This prevents polar clustering of the Tar chemoreceptor [13]. The Tar-YFP fusion was expressed from the plasmid pVS263 [13].

### FCS

The microscope for FCS measurements was essentially as described in [24] with some slight modifications. The excitation source (∼164 µW) at 514.5 nm was derived from the laser lines of an Ar^+^ laser (Spectra-Physics, Mountain View, CA), which was brought to a focus in the sample by a microscope objective (Nikon 60x, NA 1.2, water-immersion). The emission was collected by the same objective and was separated from the excitation by a dichroic mirror. The confocal volume was defined by a 100 µm pinhole. A single interference filter was used to allow only the fluorescence to pass through and reach the detector. Photon counting in real time was done by an avalanche photodiode detector (SPCM-AQR-16, Perkin-Elmer, Vaudreuil, Canada), and a fast correlator card (Correlator.com, NJ) was used to control the data collection as well as the subsequent autocorrelation analysis for the FCS measurements.

Before every experiment, the confocal microscope system was calibrated by FCS measurements of the characteristic diffusion time of R6G (Molecular Probes). For this three-dimensional diffusion in a prolate ellipsoidal Gaussian observation volume, the autocorrelation function *G*(τ) arising from diffusion of a single species can be described by (See [20]–[27] for general discussions of FCS):
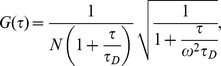
where τ is the lag time, τ_D_ is the characteristic diffusion time of the species during which it resides in the confocal observation volume, ω is the axial (*z*_0_) to lateral (*r*_0_) dimension ratio of that volume, and *N* is the mean number of fluorescent molecules in the time limit 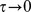. For the measurements used here, *r*_0_≈400 nm and *z*_0_ ≈2 µm. τ_D_ and the diffusion constant *D* are related by:



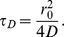



**Figure 3:**
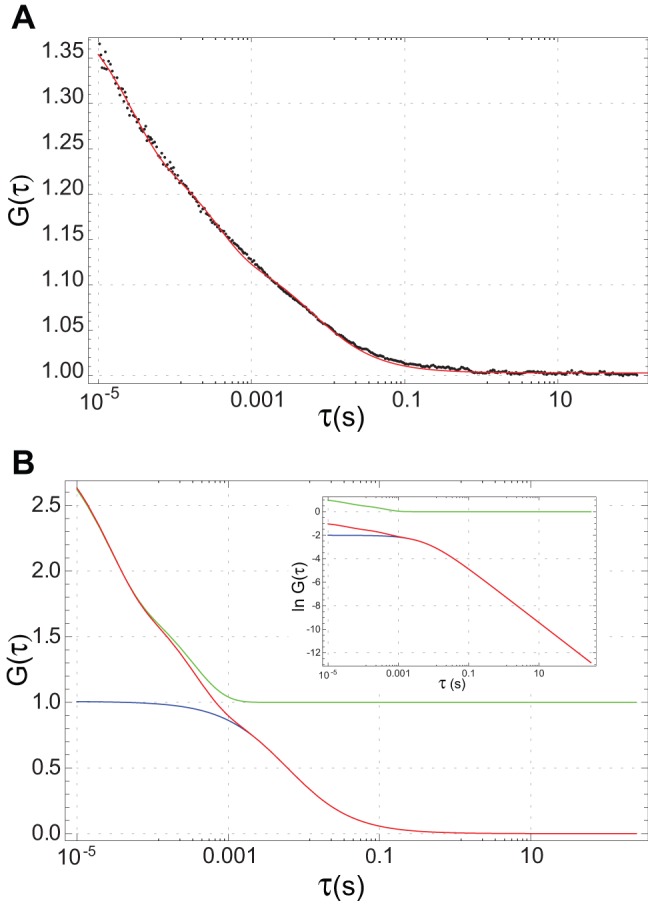
FCS autocorrelation curves. (A) A typical FCS autocorrelation curve *G*(τ) for TetA-YFP in the strain DGC103 (black dots), along with the fitted theoretical curve (red line). This curve yields *D* = 6.4 µm^2^/s. (B) The diffusion and fast dynamics terms in the fitting function for *G*(τ) (see Methods) plotted separately to illustrate how they contribute. The red curve is the entire model *G*(τ), identical to the curve in (A). The blue curve is *G_D_*(τ) + *B*, the diffusion contribution. The green curve is *G_T_*(τ)/N + *B*, the fast dynamics contribution. (B, inset) Logarithms of the contributions to *G*(τ) with the background *B* subtracted: ln(*G*(τ)-*B*) (red), ln(*G_D_*(τ)) (blue), ln(*G_T_*(τ)) (green). The three curves satisfy ln(*G*(τ)-*B*)  =  ln(*G_D_*(τ)) + ln(*G_T_*(τ)).

Each FCS curve was obtained by computing the autocorrelation function of the fluorescence intensity over a 120 second period. All of the signal traces were examined, and those with extensive drift or large spikes (less than 10% of all curves) were rejected. The remaining autocorrelation curves were fit using a form of *G*(τ) composed of a single 2D diffusion timescale and two exponential timescales that model fast photophysical dynamics (as in [51]; for derivations see [22]):
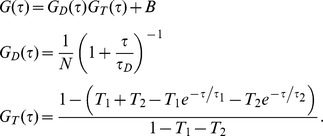



Here, the full autocorrelation function has been expressed as the product of its translational diffusion term, *G_D_*(τ), and its fast dynamics term, *G_T_*(τ), where τ_Δ_ is the 2D diffusion time constant, *N* is the average number of fluorescent molecules in the confocal volume, *B* is the overall background, and *T*_1_, *T*_2_, τ_1_ and τ_2_ are the respective amplitudes and time constants of the two fast dynamic components. All of these variables (τ_Δ_, *N*, *B*, *T*_1_, *T*_2_, τ_1_ and τ_2_) were treated as fit parameters. Nonlinear regression fits to the autocorrelation curves, beginning at lag times of 10 µs (which was limited by the instrumentation), were performed using Mathematica. See Figure 3A for an example of an autocorrelation function and fit. All FCS curves reflected the same two fast dynamic components (τ_1_ and τ_2_): one near 5–30 µs which reflects the triplet state of the fluorophore, and one near 300 µs which may reflect photophysical processes such as YFP blinking [52], [53]. Values of τ_Δ_ ranged from 3–15 ms, reflecting the diffusion of the species of interest. Figure 3B dissects the model curve that fits the data in Figure 3A into its component timescales. For lag times shorter than 1 ms, the fast dynamics terms dominate, but for longer lag times their contribution vanishes and the diffusion term becomes the greatest contributor.

### FRAP

The microscope used for FRAP is described in [54]. Briefly, photobleaching was performed with a 405 nm laser using an iLas^2^ system (Roper Scientific) with MetaMorph software (Molecular Devices) coupled to a spinning disk confocal microscope (DM4000; Leica) with a 100×1.4 NA oil immersion objective, an XY Piezo-Z stage (Applied Scientific Instrumentation), a spinning disk (Yokogawa), an electron multiplier charge-coupled device camera (ImageEM; Hamamatsu Photonics), and a 488 nm laser (LMM5; Spectral Applied Research) controlled by MetaMorph software, which was used for YFP fluorescence excitation.

Our FRAP protocol and analysis was essentially as described in [13]. The bleach region of interest (ROI) was a polar cap region ∼1.8 µm long, which was about 25% of the average cell length of 7.3 µm (range 5.2–9.4 µm). The ROI was uniformly bleached with a 336 ms laser scan at 100% intensity. Images were acquired with 100 ms exposures before and immediately after bleaching. The postbleach image series for TetA-YFP consisted of 10 images, one taken every 1 s, then 10 more, one taken every 10 s, then 10 more, one every 30 s. For cytoplasmic YFP, the image series was 10 images taken every 336 ms, then 10 images every 1 s, then 10 images every 2 s.

**Figure 4:**
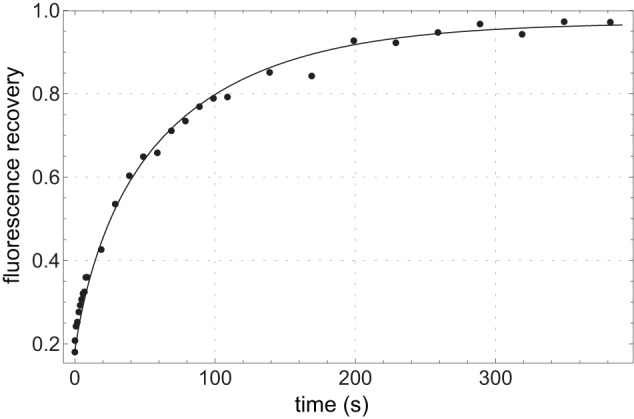
FRAP time series. A typical FRAP recovery time series (dots), following the relative TetA-YFP fluorescence in the bleached region of interest (ROI) in a cell of DGC103 after bleaching, which was calculated as described in Methods. The fitted theoretical curve (line) yields *D* = 0.101 µm^2^/s and *K* = 0.97.



Postbleach images were analyzed in ImageJ. Each cell was divided into two masks, one of the (bleached) ROI and the other of the remainder of the cell. The relative fluorescence in the ROI was computed by dividing the total fluorescence in the ROI mask by the total cell fluorescence in the same image, which also compensates for bleaching from excitation light during image acquisition. This quantity was normalized by the ratio of the ROI mask area to the total area to give a value that will recover to 1 if all fluorescent molecules in the cell are mobile (mobile fraction of 1). See Figure 4 for a typical recovery curve. The recovery curves were modeled with the one-dimensional diffusion equation as described in [13]. Briefly, we take the cell to run from *x* = 0 to *x* = *L* and the bleached region runs from *x* = 0 to *x* = *L_b_*. The fluorescence intensity as a function of position *x* and time *t* is denoted *u*(*x,t*). The recovery *R*(*t*) is approximated by:

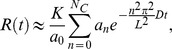
where



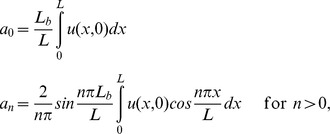



*K* is the mobile fraction, *D* is the diffusion constant, and *N_C_* is a cutoff on the sum.

The initial concentration profile *u*(*x*,0) was approximated by a piecewise linear profile derived from the first post-bleach image, consisting of two constant regimes corresponding to the bleached and unbleached areas joined by a linear transition region of 10 pixels. The data was fit to this model with parameters *K* and *D* by nonlinear regression with Mathematica. All data sets were readily fit with *N_C_*  = 4, i.e. keeping only the first four terms of the sum. Higher values of *N_C_* had no effect on the values of *D* to within one part in 1000.
